# Perception of women with classic congenital adrenal hyperplasia and their parents on genital surgery and a diagnosis of differences of sex development: a retrospective survey

**DOI:** 10.1007/s40618-025-02727-w

**Published:** 2025-10-27

**Authors:** Lea Tschaidse, Andrea Sappl, Hanna F. Nowotny, Ann-Christin Welp, Matthias K. Auer, Heinrich Schmidt, Nicole Reisch

**Affiliations:** 1 Department of Medicine IV, LMU University Hospital, LMU Munich, Ziemssenstraße 5, 80336 Munich, Germany; 2 Department of Pediatrics, Dr. von Hauner Children’s Hospital, LMU University Hospital, LMU Munich, Munich, Germany

**Keywords:** Differences in sex development, Congenital adrenal hyperplasia, Androgen excess, Atypical genitalia, Virilization, Genital surgery

## Abstract

**Purpose:**

Classic congenital adrenal hyperplasia (CAH) due to 21-hydroxylase deficiency leads to adrenal androgen excess, regularly causing prenatal virilisation of the external genitalia in affected females, commonly corrected by early genital surgery. As this practice is controversial, this study retrospectively assessed patients’ and parents’ perspective on this matter.

**Methods:**

Adult female patients with classic CAH who had undergone genital surgery and their parents participated in this single-centre, cross-sectional survey study. Patients completed the female Sexual Function Index (fSFI) and female Sexual Quality of Life (SQOL-F), while parents completed the Decision Regret Scale (DRS).

**Results:**

Among 46 patients, 45.7% had one genital surgery, while 54.3% had multiple procedures. Most (80.4%) had their first surgery by the age of five, of which seven had a second surgery by the age of five. In 95.5% of cases, the surgical decision was made by parents. Median (IQR) fSFI score was 20.3 (5.3) and the median (IQR) SQOL-F score was 97.0 (32.5). Among 22 parents, 70.5% showed no or mild regret concerning the decision for surgery, while 29.4% reported moderate to strong regret. Most patients (63.0%) and parents (86.4%) found the term “DSD” inappropriate for CAH.

**Conclusion:**

Women with classic CAH often undergo early genital surgery, with their parents being mostly responsible for treatment decisions. Despite sexual dysfunction, patients show good sexual quality of life. Most patients and parents have few or no regrets about the decision to have early genital surgery performed and are largely critical of CAH being labelled as DSD.

## Introduction

Differences of sex development (DSD) comprise a complex and diverse group of conditions characterized by atypical chromosomal, gonadal, or anatomical sex development [1]. One of the most prevalent causes of DSD, affecting approximately 1 in 7,000 to 1 in 9,000 female live births, is classic congenital adrenal hyperplasia (CAH) due to 21-hydroxylase deficiency (21OHD) in women [2]. Classic CAH due to 21-hydroxylase deficiency is an autosomal recessive disorder primarily affecting steroidogenesis in the adrenal glands, presenting with varying cortisol (simple virilising form) and, in some cases, additional aldosterone deficiency (salt wasting form), as well as ACTH driven adrenal androgen excess due to a lack of negative feedback. In the classic form, intrauterine exposure to androgen excess during critical stages of fetal development regularly causes prenatal virilization of the outer genitalia in affected girls. The degree of virilization varies in these patients, ranging from mild clitoromegaly to complete virilized external genitalia, and is classified according to Prader stages, with higher stages indicating a higher degree of virilisation [3].

So far, virilization of the outer genitalia in female patients with classic CAH has commonly been corrected by genital surgery during the early years of life. Depending on the degree of virilisation, this can include vaginoplasty, as well as labial and clitoral surgery. According to the “Consensus Statement on 21-Hydroxylase Deficiency from The Lawson Wilkins Pediatric Endocrine Society and The European Society for Paediatric Endocrinology” [4], the surgery should ideally be completed as a single-stage operation and should be performed at age 2 to 6 months or before the age of two years at the latest, according to surgical opinions, due to better tissue elasticity. Surgical goals are to create a genital appearance aligned with the child’s sex, allow normal urinary function, and support future sexual and reproductive health. In cases of mild virilisation (Prader stage < 3), surgical intervention is not recommended. Revision surgeries may be necessary in adolescence or adulthood [4–6]. However, the practice of early genital surgery has been questioned and the management of genital ambiguity in patients with DSD and especially female patients with CAH has been a subject of extensive debate, leading to significant controversy in this field.

Whilst some professionals are in favour of early surgery with the goal to facilitate gender development, reduce the children’s risk for intersex-stigmatisation and also parental anxiety [7, 8], there has been an increased demand to postpone genital surgery under consideration of fundamental human rights, if it is not immediately medically necessary, until the child concerned reaches the age of consent. One of the main arguments against early surgery is denying patients the right to participate in irreversible decisions regarding their anatomy and gender and therefore eliminating the possibility of an open future [9]. In context of this ongoing debate, Germany passed a law on “Treatment of Children with Variants of Gender Development” in May 2021, which prohibits surgeries with the sole aim to aesthetically align external genitalia to a specific gender or sex in newborns and children before the age of consent. In DSD conditions, the law states that surgical interventions aimed at aligning the appearance of the external genitalia with the assigned sex can only be consented to by parents if the procedure is urgently needed to prevent serious harm to the child’s life or health. Otherwise, such procedures must be postponed until the affected children can participate in the decision-making process themselves or it requires approval from the family court [10].

This raises another important question which is to whether CAH should be classified as a DSD and therefore be subject to this law. Previous studies, along with the perspective of affected individuals and their families imply that they do not consider CAH to be part of the DSD spectrum and are in favour of early genital surgery [11–13]. Therefore, the aim of this study was to retrospectively assess the perspective of female patients with classic CAH and their parents from a single Germany center on genital surgery in CAH and the diagnosis of DSD and to further assess the psychosexual outcome of these patients.

## Methods

### Participants

Adult female patients with a confirmed diagnosis of classic CAH who had undergone genital surgery at least once in their lives, and parents were enrolled in this single-centre, cross-sectional, survey study. All participants were recruited at the endocrine outpatient clinic at the university hospital of Munich, LMU, Germany.

### Questionnaire

The data collection was conducted using questionnaires. In addition to collecting sociodemographic data, the questionnaires also included specific sections for both the patients and the parents. The patient’s questionnaire included the female Sexual Function Index (fSFI), a comprehensive self-reporting tool, consisting of 19 items, which evaluates various aspects of sexual function in women in the preceding four weeks. Each item is rated on a five-point Likert scale, with 15 items including an additional response option of zero, indicating no sexual activity in the past four weeks. The subscales of the fSFI, comprising the six domains desire, arousal, lubrication, orgasm, satisfaction and pain, are scored individually. A maximum score of six for each subscale can be reached, which reflects the average performance or functioning in the respective domain. The total fSFI score can range from 2 to 36, providing an overall measure of sexual function, with lower scores indicating lower levels of sexual functioning. A total score of ≤ 26.55 indicates sexual dysfunction [14].

The patient’s questionnaire also contained the female Sexual Quality of Life (SQOL-F), a self-reported assessment tool designed to evaluate the effect of sexual dysfunction on women’s quality of life [15]. It consists of 18 items, each rated on a 6-point scale from “completely agree” to “completely disagree,” resulting in a total score range of 18–108. Higher scores indicate a better sexual quality of life [16]. Although there is no official cut-off, it has been suggested, that a total score of ≥ 85 indicates good SQOL, a total score between 52 and 84 indicates moderate SQOL and a total score of ≤ 51 indicates poor SQOL [17, 18].

The parent’s questionnaire included the Decision Regret Scale (DRS), which assesses feelings of remorse or distress regarding a medical healthcare treatment decision through five statements. Respondents indicate their level of agreement or disagreement by selecting a number from one (“strongly agree”) to five (“strongly disagree”). Scores range from 0 to 100, with higher scores reflecting greater levels of regret (DRS 0 = no regret; DRS 1–25 = mild regret; DRS ≥ 26 = strong regret) [19].

### Statistical analysis

Concerning the fSFI, the SQOL-F and DRS, missing values led to exclusion. Data were tested for normality using the Kolmogorov-Smirnov test. Normally distributed data are given with mean and standard deviation (SD), non-normally distributed data are presented as median and interquartile range (IQR). For correlation analysis, Spearman’s correlation coefficient for non-parametric data was calculated. A p-value of < 0.05 was considered statistically significant. For statistical analysis and graphical representation, SPSS version 29.0 and Prism 9 were used.

## Results

### Female CAH patients

A total of 48 patients with classic CAH were recruited for this study with 2 patients being subsequently excluded from analysis due to missing data. Therefore, the final study population contained 46 patients of female sex (i.e. 46, XX) with a median age of 31 years (18–66). A total of 29 (63.0%) presented with salt wasting (SW-) and 17 (37.0%) with simple virilising (SV-) CAH. 11 patients (23.9%) reported at least one psychiatric diagnosis. Among them, depression was the most common, affecting nine patients, followed by anxiety disorder and borderline personality disorder, both affecting two patients each. Psychosis, ADHD, binge-eating disorder, sleep disorder, and intellectual disability each affected one patient in the study cohort. All patients identified as female. Patient characteristics are depicted in Table 1.

**Table 1:** Basic characteristics of included patients with classic CAH

		*N*	Total *N*	%
Number of patients			46	
Median age at assessment (min-max) in years	31 (18–66)		46	
Nationality			46	
German		43		93.5
Turkish		1		2.2
Austrian		1		2.2
N/A		1		2.2
Highest degree of education			46	
University degree		14		30.4
Vocational training		13		28.3
School education				
High		5		10.9
Middle		9		19.6
Low		2		4.3
N/A		3		6.5
CAH form			46	
SW		29		63.0
SV		17		37.0
Prader stage at birth			46	
2		1		2.2
3		11		23.9
4		10		21.7
5		4		8.7
N/A		20		43.5

CAH, congenital adrenal hyperplasia; SW, salt-wasting; SV, simple-virilizing

**Fig. 1:**
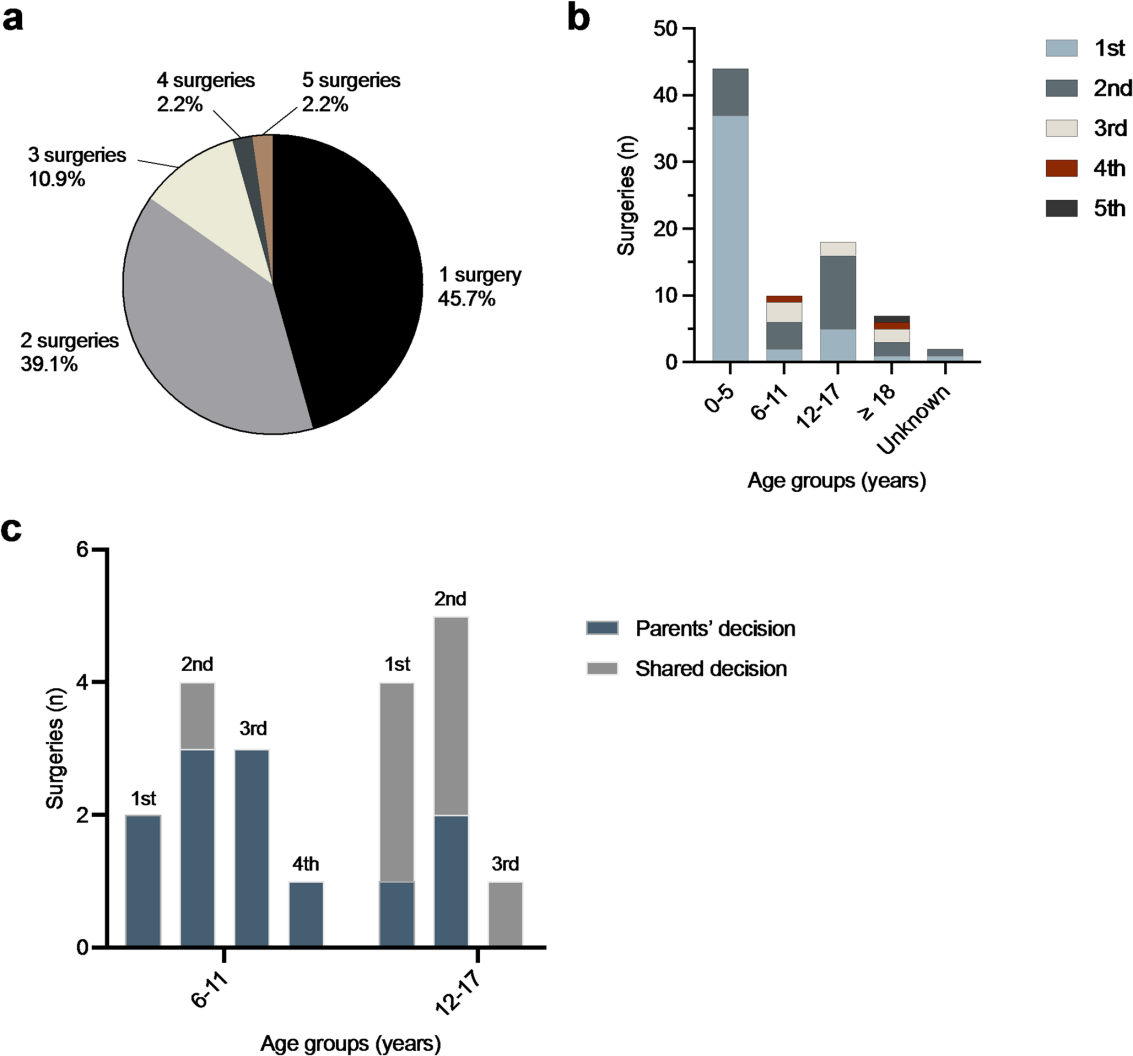
Displayed is the frequency of received genital surgeries of included patients with classic CAH during the course of their lives (**a**) as well as the frequency of surgeries based on age and which operation (e.g., first, second, third, fourth, fifth) patients underwent within each age group (**b**). For the age groups 6–11 and 12–17 years, the decision to undergo surgery is also categorized based on the number of operations (i.e., first to fourth) and whether the decision was made by the parents or jointly (**c**)

A total of 81 genital surgeries were reported in our study sample. About half of the patients (*n* = 21; 45.7%) had undergone one genital surgery in their life, the other half (*n* = 25; 54.3%) more than one (Fig. 1a). Most surgeries (*n* = 44; 54.3%) were performed up to the age of five years, of which 37 (84.1%) were first and seven (15.9%) were second surgical interventions. Between 6 and 11 years of age, 12.3% (*n* = 10) of surgeries were performed (two first surgical intervention, four second surgical intervention, three third surgical intervention, one fourth surgical intervention). Another 22.2% (*n* = 18) of surgeries were performed at 12–17 years (five first surgical intervention, 11 second surgical intervention, two third surgical intervention) and 8.6% (*n* = 7) at 18 years and older (one first surgical intervention, two second surgical intervention, two third surgical intervention, one fourth surgical intervention, one fifth surgical intervention). In 2.5% of cases (*n* = 2) the age was not documented. The frequency of surgeries according to age groups and in which age group each respective operation (e.g., first, second, third, fourth or fifth) was performed is shown in Fig. 1b. A total of 48.9% (*n* = 23) of patients reported that they were satisfied with the timing of the first operation, 29.8% (*n* = 14) of the patients would have favoured an earlier and 2.1% (*n* = 1) a later timing, while 17.0% (*n* = 8) did not have an opinion. Of those favouring an earlier timing for the first operation, seven had their first operation up to the age of five years, two between 6 and 11 years, four between 12 and 17 years and one at 18 years and older. The patient favouring a later timing had her first operation up to the age of five years. The patient classified as Prader stage 2 underwent one genital surgery in her life at the age of 16 years. A total of 40.4% (*n* = 19) of the patients reported being able to remember the surgeries, while 53.2% (*n* = 25) had no memory of them (Unknown: 6.4%; *n* = 3). 46.8% (*n* = 22) of patients stated that they were very satisfied with the aesthetic result of the operations, 29.8% (*n* = 14) were satisfied, 14.9% (*n* = 7) were less satisfied and 4.3% (*n* = 2) were dissatisfied (Unknown: 4.3%; *n* = 2). 55.3% (*n* = 26) of patients reported that they were very satisfied with the functional outcome of the operations, 31.9% (*n* = 15) were satisfied, 4.3% (*n* = 2) were less satisfied and 4.3% (*n* = 2) were dissatisfied (Unknown: 4.3%; *n* = 2). According to the patients, taking into account all reported surgeries, in the majority of cases (*n* = 54; 66.7%), the decision to undergo surgery had been made by their parents, in 7.4% (*n* = 6) of cases by the patients and in 12.3% (*n* = 10) of cases together. In 2.5% (*n* = 2) the patients stated that the decision was made by medical staff and in 11.1% (*n* = 9) of cases the patients did not specify who made the decision. Considering the different age groups and the respective surgical procedures, decisions for operations between ages 0–5 (only first and second surgeries) were made almost exclusively by parents (*n* = 42), with medical staff making the decision in two cases. In the age group of 18 years and older (covering first to fifth surgeries), most decisions were made independently by the patients (*n* = 6), with one case involving shared decision-making with the parents. Surgical decision-making for the age groups 6–11 and 12–17 years is presented in Fig. 1c. When asked about the patients’ satisfaction about their parents’ decision for surgery, 76.6% (*n* = 36) stated that they were satisfied, whilst 25.5% (*n* = 12) were not satisfied. 8.5% (*n* = 4) did not have an opinion on this matter and 8.5% (*n* = 4) did not answer the question. However, 78.7% (*n* = 37) of patients would have made the same decision as their parents and 8.5% (*n* = 4) would have chosen differently. 6.4% (*n* = 3) did not have an opinion on this matter and 6.4% (*n* = 3) did not answer the question. In cases where patients participated in the decision-making process or made the decision independently, various reasons for undergoing surgery were reported. These reasons are categorized by age group and presented in Table 2. When asked if the patients would have been happier without surgery in childhood and/or adolescence, 70.2% (*n* = 33) patients disagreed, 17.0% (*n* = 8) patients somewhat disagreed, 2.1% (*n* = 1) patients somewhat agreed and 2.1% (*n* = 1) fully agreed (Unknown: 8.5%; *n* = 4). Concerning the patients’ relationship to their parents in childhood, 55.3% (*n* = 26) stated that their relationship was very good, 29.8% (*n* = 14) stated that the relationship was good, whilst 6.4% (*n* = 3) had a less good relationship and 2.1% (*n* = 1) had a bad relationship.

**Table 2:** Given reasons for the decision to undergo surgery, according to age groups, in cases in which patients participated in the decision-making process or made the decision independently

	*N*	Reasons for surgery
Age group (years)		
6–11	1 1 1 1	Due to doctors’ recommendations To facilitate the process of finding one’s own gender identity To facilitate social interaction with other people To improve sexual functioning
12–17	5 1 2 5 1	Due to doctors’ recommendations To facilitate the process of finding one’s own gender identity To facilitate social interaction with other people To improve sexual functioning To reduce the occurrence of urinary tract infections
≥ 18	1 1 3 1	Due to doctors’ recommendations To facilitate social interaction with other people To improve sexual functioning To restore a “normal state
Unknown	1	Due to doctors’ recommendations

Concerning psychosexual aspects, 60.9% (*n* = 28) of patients were in a relationship at the time of data collection (single: *n* = 16; 34.8%; Unknown: *n* = 2; 4.3%). 78.3% (*n* = 36) identified as heterosexual and 15.2% (*n* = 7) as homo- or bisexual (Unknown: 6.5%; *n* = 3).

**Fig. 2:**
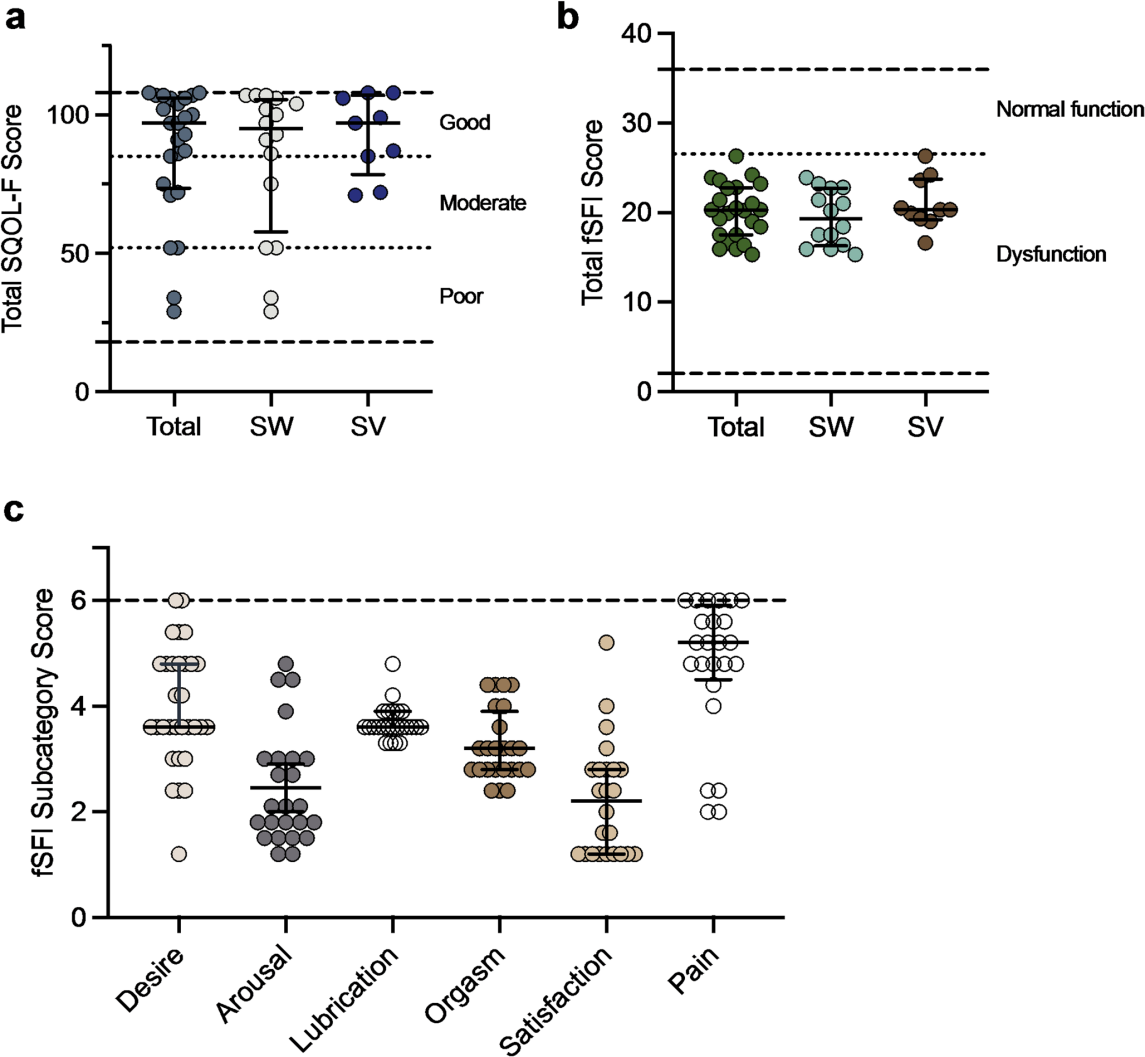
Depicted are the scores of the female sexual Quality of life Questionnaire (SQOL-F) (range: 18–108) divided into good, moderate and poor SQOL-F (**a**) and the scores of the female sexual functioning index (fSFI) (range: 2–36; sexual dysfunction with a score < 26.55) (**b**), including the fSFI subcategories (maximum score: 6) (**c**), of all included patients with CAH. CAH, congenital adrenal hyperplasia; SW, Salt-wasting; SV, simple-virilizing

The SQOL-F was completed by a total of 31 patients, of which 6 were excluded from further analysis due to missing values. The remaining 25 patients scored a median (IQR) SQOL-F of 97.0 (32.5). 18 (72.0%) patients achieved a score of 85 or higher, which corresponds to a good SQOL, while five (20.0%) achieved a moderate and two (8.0%) a poor SQOL. There were no significant statistical differences between SW and SV patients concerning SQOL-F (95.0 (47.8) vs. 97.0 (28.5); *p* = .637) (Fig. 2a) and no statistical difference in mean (SD) SQOL-F Score compared to healthy control subjects from the literature (87.0 (23.4) vs. 85.0 (18.8); *p* = .693) [20].

The fSFI was completed by 31 patients. In seven out of 31 of the included patients, only the “desire” section of the fSFI could be evaluated due to the absence of sexual intercourse in the preceding four weeks of the study. The total median (IQR) fSFI score (*n* = 24) was 20.3 (5.3). There were no significant statistical differences between SW and SV patients in regard to total median (IQR) fSFI score (19.3 (6.5) vs. 20.3 (4.5); *p* = .259) (Fig. 2b). All patients only achieved a maximum fSFI score of < 26.55, which corresponds to sexual dysfunction. Compared to healthy control subjects from the literature [20] our patients showed significantly lower mean (SD) fSFI scores (20.1 (3.0) vs. 25.7 (7.3); *p* < .001). The scoring in the subcategories of the fSFI is displayed in Fig. 2c.

The fSFI and SQOL-F Score showed a significant negative correlation (*r* = − .439; *p* = .036).

### Parents of children with CAH

A total of 22 parents of a child with classic CAH were included for this study, with their general characteristics presented in Table 3. Fourteen of the children (63.6%) of the surveyed parents had one genital surgery in their lifetime, eight (36.6%) had two or more resulting in a total of 29 operations. With 22 operations (75.9%), the majority were performed during infancy (0–5 years), three (10.3%) during childhood (4–12 years) and four (13.8%) during adolescence (13–17 years). 12 parents (54.4%) were very satisfied with the course of the surgeries, eight (36.4%) were satisfied, two (9.1%) were less satisfied whilst none were dissatisfied. Fifteen (68.2%) were very satisfied with the timing of operation, three (13.6%) were satisfied, one (4.5%) was less satisfied and three (13.6%) were dissatisfied. A total of 18 parents (81.8%) would make the same decision again today, two (9.1%) were uncertain and two (4.5%) would choose differently (4.5%), with one preferring an early timing of surgery. In response to the question of whether the daughter had ever expressed reproach regarding the parent’s decision for the surgery, 21 parents (95.5%) answered “no” and only one (4.5%) answered “yes” with the remark “occasionally”.

**Table 3:** Basic characteristics of included parents

		*N*	Total *N*	%
Number of parents			22	
Median age at assessment (min-max) in years	59 (47–80)		22	
Sex			22	
Female		20		90.9
Male		2		9.1
Nationality			22	
German		19		86.4
Turkish		1		4.5
Italian		1		4.5
N/A		1		4.5
Highest degree of education			22	
University degree		6		27.3
Vocational training		8		36.4
School education				
High		3		13.6
Middle		2		9.1
Low		1		4.5
Primary School		1		4.5
N/A		1		4.5
CAH form of the child			22	
SW		13		59.1
SV		9		40.9

CAH, congenital adrenal hyperplasia; SW, salt-wasting; SV, simple-virilizing

A total of 17 parents completed the DRS. Of these, nine parents (52.9%) showed no regret (DRS 0) concerning the decision of surgery, three (17.6%) showed mild regret (DRS 1–25) and five (29.4%) showed moderate to strong regret (DRS ≥ 26).

### Medical term of diagnosis

Both the patients’ as well as the parents’ questionnaire contained questions regarding the medical terminology of the diagnosis. The results for both cohorts are presented in Table 4.

**Table 4:** Patients’ and parents’ responses concerning the medical terminology of the diagnosis

	Response	Patients, total *n* = 46 *N *(%)	Parents, total *n* = 22 *N *(%)
Question			
Would you prefer the translation of the English term “congenital adrenal hyperplasia” (CAH) rather than the German term “Adrenogenitales Syndrom” (AGS) to describe your or your daughter’s diagnosis?			
	Yes	21 (45.7)	10 (45.5)
	No	12 (26.1)	9 (40.9)
	Uncertain	9 (19.6)	3 (13.6)
	N/A	4 (8.7)	0 (0.0)
Have you heard the term “difference/disorder of sex development” (DSD) before?			
	Yes	16 (34.8)	11 (50.0)
	No	25 (54.3)	11 (50.0)
	Uncertain	3 (6.5)	0 (0.0)
	N/A	2 (4.3)	0 (0.0)
Do you consider the term “difference/disorder of sex development” (DSD) appropriate for CAH?			
	Yes	8 (17.4)	1 (4.5)
	No	29 (63.0)	19 (86.4)
	Uncertain	6 (13.0)	2 (9.1)
	N/A	3 (6.5)	0 (0.0)
What impact does the term “difference/disorder of sex development” (DSD) have on individuals with CAH?			
	Positive	0 (0.0)	0 (0.0)
	Negative	34 (73.9)	18 (81.8)
	None	8 (17.4)	2 (9.1)
	N/A	4 (8.7)	2 (9.1)
Do you find the term “Intersex” appropriate for yourself/your daughter?			
	Yes	3 (6.5)	1 (4.5)
	No	38 (82.6)	20 (90.9)
	Uncertain	2 (4.4)	1 (4.5)
	N/A	3 (6.5)	0 (0.0)
Do you consider the condition as…			
	Adrenal disease	41 (89.1)	21 (95.4)
	DSD	0 (0.0)	1 (4.5)
	Both	3 (6.6)	0 (0.0)
	N/A	2 (4.3)	0 (0.0)
What term do you use when talking about the condition in your social environment?			
	Adrenal disease	38 (82.6)	17 (77.3)
	DSD	0 (0.0)	0 (0.0)
	Different term	5 (10.9)*	5 (22.7)**
	N/A	3 (6.5)	0 (0.0)
Would you prefer to rename AGS to CAH?			
	Yes	19 (41.3)	8 (36.4)
	No	16 (34.8)	8 (36.4)
	Uncertain	8 (17.4)	4 (18.2)
	Other	0 (0.0)	1 (4.5)***
	N/A	3 (6.5)	1 (4.5)

*N = 2 do not speak about the condition; Otherwise used terms: *n* = 1 “comparable to diabetes mellitus”, *n* = 1 “cortisol deficiency”, *n* = 2 “metabolic disease”

**N = 2 do not speak about the condition; Otherwise used terms: *n* = 1 “cortisol deficiency”, *n* = 2 “metabolic disease”

***N = 1: “equal”

CAH, congenital adrenal hyperplasia; AGS, Adrenogenitales Syndrom; DSD, difference/disorder of sex development

## Discussion

Our data showed that in both our cohorts - patients and parents - the majority of genital surgeries were performed up to the age of five years and therefore at a time, when the children themselves were not able to consent due to the lack of understanding of the matter. The majority of patients in our study (53.2%) were too young to even remember the surgery. Therefore, in our patient cohort, in nearly two thirds of documented surgeries the parents had made the decision to undergo surgery. This is certainly also influenced by the fact that in the case of strongly virilised genitals, operations such as the separation of a common urogenital sinus in some cases may be necessary to prevent infections of the urogenital tract in the early days of life. However, it is a critical point, as early surgery at a time when the child itself is not yet capable of giving consent and thus cannot make the decision to have genital correction is currently being discussed very controversially [7, 9]. Although the decision had been made without their consent, the majority of patients were very satisfied with their parent’s decision and would have made the same decision as their parents. Nearly half of the patients were satisfied with the timing of the operation and about 30% would even have favoured an earlier timing of surgery. Only one patient would have wished for a later timing in life concerning the operation. In the parent’s cohort we found similar data, with the majority of parents being satisfied or very satisfied with the course of the surgery as well as timing of the operation. Also, more than 80% of parents, would make the same decision again today. This is in line with data from a multicentre European study including 459 patients with DSD, in which the majority of female and male patients with CAH and XY DSD with androgen effect stated, that the decision about surgical procedures should not be postponed until the age of consent and that the preferred timing for surgery is infancy and childhood and therefore between one month and 12 years of age [12]. In an American survey study with 41 women with CAH due to 21-hydroxylase deficiency, when asked about the preferred timing of genital surgery, the majority stated infancy and early childhood [13]. In a Finish questionnaire-based study including 24 female patients with a DSD, who had undergone genital surgery in childhood including clitoral reduction and/or vaginal reconstruction, 17 believed that the timing of the surgery had been appropriate and three thought the timing even too late, while none thought it was too early [21]. In a recent retrospective analysis from Sweden, including 62 women with CAH due to 21-hydroxylase deficiency, with about 40% each, the majority of women also stated that they favoured early surgery (≤ 4 years of age) or had no preference, whilst 19% of the women surveyed were in favour of later surgery (≥ 10 years of age) [22]. In a study from China on the long-term outcome of genitoplasty in DSD with partially late clinical presentation and therefore late surgical intervention, 74% of the cohort who underwent surgery in adolescent at a median age of 14.2 years and 78% of the cohort who underwent surgery in adulthood at a median age of 23.2 years thought the timing of surgery to be late [23].

With a mean age of 31 years, our study comprises a rather young sample of women with CAH, who have been aware of the current public discussion regarding the management of genital surgery in children with DSD and the increasing demand for self-determination and therefore to postpone surgery and wait until the children are able to partake in the decision. Still, the majority of patients and parents clearly state that they favour early surgery. In a French case-control study, about 90% of patients with CAH and 100% of parents stated, that genital surgery should be performed in the first year of life. In contrast, only 50% of the control patients and about 65% of the control parents stated that genital surgery should take place in the first year of life, while the rest were predominantly in favour of surgery in puberty. The main reason given for this was, that the “decision should be made by the child”, “to avoid making mistakes” and “to avoid the responsibility in case of problems down the road” [24]. This difference in the results also clearly highlights the different opinions between those affected themselves by CAH and the general population on that matter, especially with the fear of non-affected individuals that an early decision without the consent or involvement of patients with CAH concerning genital surgery could cause irreversible harm to the person concerned.

All of the 46,XX CAH patients included had a female gender identity, which is in line with previous studies, in which the vast majority of 46,XX CAH patients identified as female [25]. One argument against early genital surgery is the potential for gender dysphoria in adolescence or adulthood through incorrect gender/sex rearing and upbringing in newborns and children. However, studies have reported a relatively low prevalence of gender identity disorder among CAH patients so far [26–28]. A meta-analysis by Babu, Shah [29] even showed that gender identity disorder was significantly more prevalent in 46,XX CAH patients raised as male (15%) compared to those raised as female (4%), clearly supporting the recommendation for female sex and gender assignment of these patients at birth. Nevertheless, there are some reports of fully masculinized 46, XX CAH patients, which equals Prader stage five, who were reared as male and did not experience gender identity disorder in adulthood [30], suggesting that male rearing may be appropriate in select cases [29]. Concerning sexual orientation, 78.3% of our patients were heterosexual, which is the same magnitude as in previous studies [25] and the majority were in a relationship at time of data collection.

More than half of the patients in our cohort underwent more than one surgery in their life, which shows the complexity of surgical reconstruction. Objective assessment of sexual function in our cohort indeed showed that all included patients met the criteria for sexual dysfunction with significantly lower scores compared to healthy control subjects. This is in line with previous data, indicating sexual dysfunction to be more frequent in female patients with CAH, compared to controls [31]. It should be noted, however, that in various healthy control cohorts reported in the literature [20, 32], the mean fSFI also fell within the range of sexual dysfunction. This suggests that the presence of sexual dysfunction might not be solely attributed to CAH. We also found a significant negative correlation between the SQOL-F and the fSFI, indicating better sexual function to be associated with better sexual QOL. Interestingly however, apart from objective function, 72% of our patients showed good and 20.0% moderate sexual quality of life, emphasizing the difference between objectively determined criteria and the personal experience of the patients, which predominates in relation to the general outcome. Overall, our cohort showed a very positive outcome with most of the patients being very satisfied and satisfied with the functional and aesthetical results after genital surgery.

Deciding whether or not to proceed with genital corrective surgery for their child is a complex and emotionally challenging process for many parents. In our cohort about 70% of parents showed no to mild regret, concerning the decision for their child to undergo genital surgery and the majority would make the same decision again today, which is comparable to an American study, in which the majority of caregivers showed no to mild regret [33]. Still, nearly 30% of parents in our study showed moderate to strong regret, which could potentially be improved through better support of the parents and caregivers in the decision-making process by a multidisciplinary team.

Concerning terminology, most of the patients and parents stated that the term DSD or intersex is not appropriate for CAH and that the term DSD has a negative impact on patients with CAH. Also, the majority of patients would prefer a renaming of “Adrenogenitales Syndrom” to the translation of the English term congenital adrenal hyperplasia. Most patients and parents classified CAH rather as a disease of the adrenal gland than a DSD. Disease of the adrenal gland better describes the pathogenesis, also as male patients do not have an alteration of the outer genitalia and the main characteristic of the disease is cortisol deficiency, not differences in sexual development nor gender identity challenges. A similar outcome was observed in an American survey of 589 patients with CAH and relatives, in which 83.6% of participants reported not identifying with the term DSD. Further 71.0% expressed dislike or strong dislike for the term, and 76.0% felt that it had a negative impact on individuals with CAH. The term was labelled misleading and patients and their relatives were worried about stigmatization [11]. Although according to a study by Davies, Knight, Savage, Brown, Malone [34], the term DSD was the preferred term over “intersex” by parents with children with DSD, only a minority thought it to be an acceptable alternative. Additionally, some expressed concern that using DSD shifts focus away from critical issues like life-threatening adrenal insufficiency, and male patients or patients with non-classic CAH often feel incorrectly linked to the term [11].

However, current data show that there are always patients who regret undergoing surgery and express dissatisfaction with the outcome [21]. Although this seems to be the minority, especially in patients with CAH when looking at the data so far, they should not be overruled by the majority. Rather it shows the complexity of this matter and that a much more sophisticated approach to the topic is necessary. Each case should be considered individually, taking into account all factors such as the exact diagnosis, degree of virilisation, the social environment, as well as medical and psychological opinions. Together with the parents and also the child, if already at an age of consent, a decision should be made. However, a legally prescribed regulation does not seem to do justice to the complexity of the situation and, in particular, does not take sufficient account of the patient’s voice. With regard to CAH, the main question here is of course the extent to which its inclusion in the DSD group is justified. The current practice of postponing surgery until an age at which the patient can give consent must be closely monitored to evaluate possible benefits but also possible negative consequences. As we do not know the outcome of patients without genital surgery in early years of life, postponing surgery may ultimately harm affected patients, for example through stigmatization due to virilized genitalia, as reported before [35].

Limitations of our study are the relatively small sample size and that only women with CAH who had undergone genital surgery were included – as there were no patients with classic CAH in our cohort not having undergone surgery. Therefore, the opinion of affected women who had not undergone genital surgery cannot be evaluated on that matter. This is especially important, as previous studies showed a trend where patients with DSD, who underwent genital surgery in childhood themselves are more likely to approve of it [12]. Another limitation of the study is the retrospective design, which also led to incomplete clinical data, such as missing surgical details and Prader stages. However, all patients were diagnosed with classic CAH clinically due to virilized external genitalia and/or salt wasting crisis and confirmed biochemically. All were treated in a single centre seen by only two different paediatric endocrinologists during the entire time period covered by our study. In our centre the standard practice was to advise patients with Prader stages 3 and higher for surgical intervention. Therefore, we can practically exclude that patients with Prader stages 1 and 2 have undergone surgery in infancy or childhood.

Our study also offers several strengths, such as the monocentric design, the inclusion of patients with CAH with different age groups, as well as the inclusion of parents. Additionally, the data was collected during the ongoing public debate, including the passing of the new law concerning the treatment of children with DSD in Germany.

## Conclusion

Our findings demonstrate that the majority of women with CAH prefer early genital surgery and are satisfied with their parents’ decision for early surgery. Although they formally present with sexual dysfunction in adulthood, they mostly have a good sexual quality of life, indicating a positive outcome. Also, the parents mostly show no regret concerning their decision for their children to undergo surgery. Furthermore, our data show that neither the patients themselves nor their parents see CAH as part of the DSD group and thus demand a different wording and also approach, especially with regard to genital surgery. As CAH is a complex disorder that affects multiple aspects of life, posing significant challenges for affected children and adult patients, as well as their parents, a multidisciplinary approach to care is essential, with a particular focus on psychological support. Comprehensive and ongoing counseling should be offered to patients and their families throughout different stages of life to address their evolving needs, particularly regarding sex- and gender-related issues.

## Data Availability

The original data presented in this study are available on reasonable request from the corresponding author.
